# Supplementary material for: AI-guided patient stratification improves outcomes and efficiency in the AMARANTH Alzheimer’s Disease clinical trial
Source: Nat Commun. 2025 Jul 17;16:6244. doi: 10.1038/s41467-025-61355-3 (PMC12271323; doi:10.1038/s41467-025-61355-3)
Supplement: Supplementary file 1 — Supplementary Information [file 41467_2025_61355_MOESM1_ESM.pdf]

Supplementary Material

Supplementary Methods

PPM training and test samples

We used data from: 1) a research cohort (The Alzheimer's Disease Neuroimaging Initiative, ADNI) for PPM training, within-sample cross-validation (n = 256) and independent test (n = 419), 2) AstraZeneca Randomized Clinical Trial cohort as independent test dataset for out-of-sample validation: Amaranth (n = 1354).

Alzheimer's Disease Neuroimaging Initiative

We trained the PPM on baseline (defined as the date of Florbetapir, (FBP) PET scan) data (n = 256) from the Alzheimer's Disease Neuroimaging Initiative (ADNI) 2/GO cohort, following our previous work [1]. We determined two classes for training the algorithm defined by baseline and longitudinal syndromic labels from clinical diagnosis independent of biomarker status, with baseline defined as the evaluation closest to the first FBP PET scan acquired in ADNI: (a) Clinically Stable (n = 100; 18 Aß+ at baseline, APOE 4(+/-)=21/79, Age mean = 73.7+-std = 6.3 years, Education mean = 16.7+-std = 2.7 years, Sex (M/F) = 51/49): CN individuals who remain stable for 4+ years following baseline (mean = 5.7+-std = 1.1 years), (b) Clinically Declining (n = 156; 130 Aß+ at baseline, APOE 4(+/-) = 95/61, Age mean=74.9+-std = 7 years, Education mean = 15.9+-std = 2.7 years, Sex (M/F) = 88/68): individuals have a baseline diagnosis (at date of FBP scan) of either CN (n = 17) or MCI (n = 139) but received a diagnosis of dementia in future clinical evaluation (i.e., progressed to dementia (n = 75)), or had been diagnosed with dementia in a clinical evaluation prior to baseline (i.e., reverted (n = 81)). We included individuals in the Clinically Declining group who were MCI at baseline but have received a diagnosis of dementia prior to baseline (i.e., reverted) in this group as we anticipate

they are likely affected by AD pathology but are at an earlier stage of AD than the Alzheimer's Clinical Syndrome (i.e., late AD) group. For all individuals, MRI data were collected across multiple acquisition sites with 1.5T and 3T scanners (GE, Philips, Siemens scanners). We regressed out age, sex, and education from the training features (MTL GM density, Aβ burden, APOE4) to account for potential confounding differences in these covariates across classes.

To validate the PPM longitudinal predictions on data that were not included in model training, we selected an independent validation sample (n = 419) including individuals with normal cognition (CN, n = 119), Mild Cognitive Impairment (MCI, n = 150; i.e. individuals who consistently received MCI diagnosis withing a period of 3 years) and Alzheimer's Disease (AD, n = 150).

AMARANTH

AMARANTH is a phase 2/3, multicenter, randomized, double-blind, placebo-controlled, global clinical trial of Lanabecestat target[2]. Patients who completed AMARANTH could elect to enter a separate 104-week delayed-start (DS) extension study, AMARANTH-EXT. AMARANTH formally entered into phase 3. Lanabecestat is a brain-permeable inhibitor of human Beta-site amyloid precursor protein-cleaving enzyme 1 (BRACE1/β-secretase). It was developed for the modification of the clinical course of AD by slowing disease progression in patients diagnosed with early AD (i.e. patients with mild cognitive impairment, MCI due to AD) and patients diagnosed with mild dementia of the Alzheimer's type. BRACE1 is a type I transmembrane aspartic acid protease related to the pepsin and retroviral aspartic protease families. BRACE1 cleaves amyloid precursor protein (APP) at the β-secretase site, and APP is then cleaved by γ-secretase generating Aβ peptides. Based on its key role in the amyloid cascade, BACE1 was considered as promising therapeutic target for slowing disease progression in AD by preventing the generation of Aβ peptides and, consequently, reducing the detrimental effects of Aβ toxicity and the formation of amyloid plaques in the brain. As

potent inhibitor of BACE1, lanabecestat is a potential disease-modifying therapy for the treatment of AD. Lanabecestat has been shown to reduce Aβ1-40 and Aβ1-42 in mice, rats, guinea pigs, dogs, and humans. At sufficient exposures, lanabecestat reduces Aβ levels in the brain and cerebrospinal fluid (CSF).

The study included a 60-day screening period and a safety follow-up 4 to 6 weeks after treatment. The protocol, patient information, consent form, and other relevant study documentation were approved by the ethics committees or institutional review boards of each site before study initiation. The study was conducted in accordance with ethical principles originating from the Declaration of Helsinki and was consistent with good clinical practice and applicable regulatory requirements. Before enrollment, all patients provided written informed consent. The trial protocols are available in [2]. This study followed the Consolidated Standards of Reporting Trials (CONSORT) reporting guidelines. Patients with a florbetapir positron emission tomography (PET) scan or CSF sample conducted as part of the study screening process could participate in the florbetapir PET imaging and/or the CSF substudy. All randomized patients in the study also had serial magnetic resonance imaging (MRI) of the brain.

Patients, Randomization, and Blinding: An interactive web and voice response system was used to randomize patients centrally. In AMARANTH (n = 1354, placebo: 53.8% female, 20mg: 53.5% female, 50mg: 52% female) eligible patients were randomized 1:1:1 to once-daily oral doses of 20mg lanabecestat, 50mg lanabecestat, or placebo, stratified by disease status at baseline (MCI attributable to AD or mild AD).

In AMARANTH, the 20mg lanabecestat, 50mg lanabecestat, and placebo tablets were identical. Blinding of the study treatment was achieved using the double-dummy technique. In this study, all patients, investigators, and sponsor staff were blinded to treatment allocation

with limited exceptions as specified in the protocols. Study treatment was dispensed during site visits, and eligible patients were instructed to take study treatment once daily orally. Adherence to study treatment was assessed by direct questioning and by counting returned tablets at each visit. Patients who consumed at least 80% of their study treatment over the course of the study were considered to be adherent.

Outcomes: The primary objective in the study was to evaluate the efficacy of lanabecestat 20mg and lanabecestat 50mg compared with placebo in slowing cognitive decline as measured by change from baseline to the end of double-blind, placebo-controlled periods on the 13-item Alzheimer Disease Assessment Scale–cognitive subscale (ADAS-Cog13) [3]. The ADAS-Cog13 measures severity of impairment in various cognitive domains (memory, language, orientation, praxis, and executive functioning). The scale has a score range of 0 to 85 points, with higher scores indicating worse performance. The scale is analyzed as a continuous measure.

Secondary objectives included efficacy evaluations of lanabecestat vs placebo on the change from baseline to the end of the placebo-controlled treatment periods on cognitive outcomes including Clinical Dementia Rating–sum of boxes (CDR-SOB), and Mini-Mental State Examination (MMSE). In AMARANTH, a time-to-event analysis was performed to evaluate the efficacy of lanabecestat to prolong time in the current disease state. This was measured by the CDR global score.

Further, biomarker objectives included evaluation of the effect of lanabecestat on amyloid markers (CSF Aβ1-42 and Aβ1-40), amyloid burden (florbetapir PET), and hippocampal volume (MRI).

Safety and tolerability of lanabecestat were evaluated in the study using the following key assessments: spontaneously reported adverse events, laboratory tests, vital signs and body

weight, and physical examinations, including neurological examinations and electrocardiograms (ECGs). Additional safety assessments included eye examinations, dermatological examinations, MRI to examine for any possible amyloid-related imaging abnormalities, and administration of the Columbia–Suicide Severity Rating Scale (C-SSRS) to assess any potential suicidality.

Diagnostic criteria

ADNI: Diagnosis in ADNI was based on MMSE, CDR, and Logical Memory for each diagnostic category as well as Geriatric Depression Scale score less than 6. AD cases were identified based on the following criteria: a) NINCDS/ADRDA criteria for probable AD, b) clinical tests: Clinical Dementia Rating = 0.5 or 1.0, and MMSE score between 20 and 26, c) memory function tests: Abnormal memory function documented by scoring below education adjusted cut-offs on the Logical Memory II subscale (Delayed Paragraph Recall, Paragraph A only) from the Wechsler Memory Scale – Revised (≤ 8 for 16 or more years of education b. ≤ 4 for 8-15 years of education c. ≤ 2 for 0-7 years of education), d) subjective memory concern, e) others: Stability of Permitted Medications for at least 12 weeks. MCI cases were identified based on the following criteria: a) clinical tests: Mini-Mental State Exam score between 24 and 30, b) memory function tests: Abnormal memory function documented by scoring below education adjusted cut-offs on the Logical Memory II subscale (Delayed Paragraph Recall, Paragraph A only) from the Wechsler Memory Scale – Revised (< 11 for 16 or more years of education, ≤ 9 for 8-15 years of education, ≤ 6 for 0-7 years of education), c)) subjective memory concern. CN cases were identified based on the following criteria: a) clinical tests: Mini-Mental State Exam score between 24 and 30, b) memory function tests: Abnormal memory function documented by scoring below education adjusted cut-offs on the Logical Memory II subscale (Delayed Paragraph Recall, Paragraph A only) from the Wechsler Memory

Scale – Revised (9 for 16 or more years of education, >=5 for 8-15 years of education, >=3 for 0-7 years of education), c) With or without subjective memory concern.

AMARANTH: Patients with mild AD dementia were eligible to be included in the study only if they met all of the following criteria at screening [4].

General Criteria: 1. Provision of signed and dated informed consent form from patient (or legal representative if required) and from study partner prior to any study-specific procedures being performed. 2. Male or Female, aged 55 to 85 years inclusive at the time of signing the consent form.

Diagnostic criteria: 3. Patients must meet the National Institute on Aging (NIA) and the Alzheimer's Association (AA) (NIA-AA) criteria for probable AD dementia. 4. Mini-Mental State Examination (MMSE) score of 20 to 26 inclusive at screening visit. 5. For a diagnosis of mild AD dementia, patient must have a Clinical Dementia Rating (CDR) global score of 0.5 or 1, with the memory box score ≥0.5 at screening. 6. Florbetapir F 18 positron emission tomography (PET) or CSF Aβ1-42 positive by central assessor for presence of amyloid.

Contraception: 7. Women must be postmenopausal, surgically sterile, or having infertility due to congenital anomaly. A postmenopausal woman is defined as either having an intactuterus with at least 6 months of spontaneous amenorrhea or a diagnosis of menopause prior to starting hormone replacement therapy. Surgically sterile women are defined as those who have had a hysterectomy, bilateral ovariectomy (oophorectomy), or bilateral tubal ligation. 8. Men with pregnant partners should use condoms from the first day of dosing until 3 months after the last dose of study treatment and abstain for 24 hours after dose administration of the florbetapir, AV-1451 or fludeoxyglucose (FDG) PET tracer. Men with partners of childbearing potential must abstain or use condoms plus an additional effective form of contraception from the first day of dosing until 3 months after the last dose of study treatment and abstain for 24 hours after dose administration of the florbetapir, AV-1451 or FDG PET tracer. For this protocol, sexual

abstinence is defined as refraining from heterosexual intercourse during the entire period of risk associated with the study treatment. True abstinence is only acceptable when it is in line with the patient's usual and preferred lifestyle.

Concomitant Medication Criteria: 9. All medication dosing should be stable for at least 30 days before screening, and between screening and randomization (does not apply to medications discontinued)

Procedural Criteria: 10. Must have completed 6 years of formal education and/or have a history of academic achievement and/or employment sufficient to exclude lifelong intellectual disability. 11. The patient must have a reliable study partner with whom he/she cohabits or hasregular contact (combination of face-to-face visits/ telephone contact acceptable). If at all possible, the same study partner should be willing to participate in study visits to provide meaningful input into the rating scales administered in this study, where study partner input is required or be available by telephone and must have sufficient patient interaction. As guidance, the ability for a study partner to meet his/her expected responsibilities for this study would normally be possible when the study partner spends no less than 10 hours per week with the subject, divided over multiple days.12. Patient and study partner must be able to read, write, and speak the language in which psychometric tests are provided, with acceptable visual and auditory acuity (corrected). 13. Study partner must be cognitively able to fulfill the requirements of the study, in the opinion of the investigator. Patients were excluded for unstable medical conditions or medication use, significant cerebrovascular pathologic findings, or a history of vitiligo and/or current evidence of post inflammatory hypopigmentation.

MRI acquisition

ADNI: Structural MRIs for the ADNI samples were acquired at ADNI-GO, ADNI2 and ADNI3 sites equipped with 1.5T and 3T MRI scanners (GE, Philips, Siemens) using a 3D MP-RAGE

or IR-SPGR T1-weighted sequences, as described online (http://adni.loni.usc.edu/methods/documents/mri-protocols).

AMARANTH: Structural MRIs for the AMARANATH samples were acquired at different sites among Australia, Belgium, Canada, USA, France, Germany, United Kingdom, Italy, Japan, Poland equipped with 1.5T and 3T MRI scanners ('Philips', 'Siemens', 'GE', 'DicomCleaner', 'Ingenia') using a 3D T1-weighted sequences.

MRI analysis: extracting medial temporal grey matter density

All imaging pre-processing was performed using Statistical Parametric Mapping 12 in MATLAB (SPM12, http://www.fil.ion.ucl.ac.uk/spm/) following our previously published pipeline[5]. Structural images were reoriented and segmented into grey matter, white matter and cerebrospinal fluid. We used the DARTEL toolbox [6] to generate a study-specific template to which all scans were normalised. Individual grey matter segmentation volumes were normalised to MNI space without modulation. The unmodulated values for each voxel represent grey matter density at the voxel location. All images were then smoothed using a 3mm3 isotropic kernel and resliced to MNI resolution 1.5 × 1.5 × 1.5 mm voxel size.

We then generated an index of medial temporal grey matter density (MTL GM density). In particular, subspace learning— that forms the backbone of the GMLVQ methodology— cannot be directly applied in the MRI voxel space due to the prohibitive number of free parameters that would need to be inferred from the sample size used for model training. Therefore, as reported in our previous work[5], to reduce dimensionality we first performed feature construction in the whole brain T1-weighted MRI voxel space using partial least squares regression with recursive feature elimination (PLSr-RFE) on ADNI (ADNI-GO ADNI-2) data. In particular, we tested for grey matter voxels that predicted memory decline (i.e. annualized change in ADNI memory composite), iteratively removing predictors (voxels) that had weak predictive values and resulting in a bilateral cluster of voxels in MTL that predict cognitive

decline. That is, this reduced set of new orthogonal features span the voxel subspace that maximises covariance with the relevant response variable (i.e. memory decline). Using this method, we determined an ROI defined by a matrix of voxel weights in the medial temporal lobe and extracted grey matter density. This grey matter density score was shown to predict memory decline, relate to individual tau burden and discriminate stable vs. progressive patients [1,5]. PPM was trained on this reduced set of features (grey matter density score) that is obtained in a data-driven way without the need to infer a prohibitive number of free parameters. We then used this predefined ROI to extract MTL grey matter density independently from AMARANTH data that was used for testing the PPM (i.e. out-of-sample validation).

PET acquisition and preprocessing

***ADNI*:** PET imaging was performed at each ADNI site according to standardised protocols. The FBP-PET protocol entailed the injection of 10 mCi with acquisition of 20 min of emission data at 50–70 min post injection. The FTP-PET protocol entailed the injection of 10 mCi of tracer followed by acquisition of 30 min of emission data from 75–105 min post injection.

Imaging analysis -PET: FBP (florbetapir PET) Aβ: FBP data were realigned, and the mean of all frames was used to co-register FBP data to each participant's structural MRI. Cortical Standardised Uptake Value Ratios (SUVR)s were generated by averaging FBP retention in a standard group of ROIs defined by FreeSurfer v5.3 (lateral and medial frontal, anterior and posterior cingulate, lateral parietal, and lateral temporal cortical grey matter) and dividing by the average uptake from the whole cerebellum to create an index of global cortical FBP burden (Aβ) for each subject [7] Finally, we converted the SUVR to the centiloid scale [8] using the following conversion taken from the LONI website CL = (FBP SUVR × 196.9) – 196.03 (http://adni.loni.usc.edu/methods/documents/, PET Protocols: ADNI Centiloids). To assign individuals as Aß positive we used the widely published threshold for ADNI FBP; SUVR = 1.1 or CL = 22.5 [9] .

Image analysis-PET: FTP (Flortaucipir PET) tau: FTP data were realigned and the mean of all frames used to co-register FTP to each participant's MRI acquired closest to the time of the FTP-PET. FTP SUVR images were generated by dividing voxel wise FTP uptake values by the average value within a mask of eroded subcortical white matter regions [10] . MR images were segmented and parcellated into 72 ROIs taken from the Desikan–Killiany atlas using Freesurfer (V5.3). These ROIs were then used to extract regional SUVR data from the normalised FTP-PET images. Left and right hemisphere ROIs were averaged to generate 36 ROIs for further analysis. We calculated the future annualised rate of tau accumulation for each of the 36 ROIs either by taking the difference between the follow-up and baseline FTP-PET scans divided by the time interval in years from baseline (when only 2 FTP scans were taken), or fitting a linear least squares fit to 3 or more FTP-PET scans and extracting the parameter estimate for the slope of the ROI SUVR vs. time in years from baseline (when 3 or more FTP scans were taken). In the ADNI3 sample the average time between FTP-PET scans is 1.22+-std: 0.38 years with the number of follow-up FTP-PET scans n (2 FTP-PET scans) = 93, n (3 FTP-PET scans) =17, n (4 FTP-PET scans) = 5.

AMARANTH**:** The PET images acquired in the study were processed using previously mentioned methods [11]. At baseline, standard uptake value ratio (SUVR) was calculated using as a ratio of the composite summary region that is an average of 6 different cortical regions (anterior cingulate, posterior cingulate, medial orbital frontal, lateral temporal, lateral parietal, precuneus) with whole cerebellum as a reference region. However, post-baseline SUVR values were calculated using 2 different reference regions: whole cerebellum and a correction factor using atlas based white matter (AWM). The SUVR with whole cerebellum was calculated as a ratio of composite summary region to whole cerebellum as a reference region, similar to the calculation at baseline. The SUVR values using AWM correction factors were calculated by dividing the composite summary ratio by an AWM correction factor. This correction factor is

a ratio of SUV values of AWM to whole cerebellum from baseline to post-baseline. Finally, we converted SUVR to the centiloid scale [8] using the following conversion taken from the LONI website CL = (FBP SUVR × 196.9) – 196.03 (http://adni.loni.usc.edu/methods/documents/, PET Protocols: ADNI Centiloids). To assign individuals as Aß positive we used the widely published threshold for ADNI FBP; SUVR = 1.1 or CL = 22.5[9].

Predictive Prognostic Model

The Generalized Matrix LVQ

In the LVQ family of algorithms the notion of a distance (metric) in the input space plays a crucial role. It governs what input points are assigned to which prototypes. The Generalized Metric LVQ (GMLVQ) is an extension of the LVQ algorithm that besides appropriate positioning of the prototypes also learns the metric to be used in the input space that enhances the class separation. The learnt metric is determined through the corresponding metric tensor. The metric tensor is a positive definite matrix Λ that incorporates feature scaling, as well as axis rotation accounting for the interplay between original input features.

More formally, given a positive definite matrix Λ, $\Lambda > 0$, the generalized form of the squared distance between an input vector x and a class prototype w takes the quadratic form $d_A(x, w) = (x\text{-}w)^T\Lambda(x\text{-}w)$.

Positive definiteness of Λ is ensured by defining Λ as $\Omega^T\Omega$, where $\Omega \in R^{(m\times m)}$ is a learnable full-rank matrix. It is important to note that only the relative distances of input points to the prototypes are significant. Therefore, the metric tensor can be multiplied by any positive real number without affecting the classifier's performance. To address this inherent ambiguity and ensure algorithm stability, the metric tensor Λ is normalized after each learning step, so that its trace is fixed throughout the learning process.

Using the steepest descent method, the cost function to be minimized through online learning is

$$f_{GMLVQ} = \sum_{i=1}^{n} (\varphi(\mu_\Lambda(x_i))$$

where

$$\mu_\Lambda(x_i) = \frac{d_\Lambda(x_i, w^+) - d_\Lambda(x_i, w^-)}{d_\Lambda(x_i, w^+) + d_\Lambda(x_i, w^-)}$$

where φ is a monotonic function (in our case identify $\varphi(l) = l$), $d_\Lambda(x_i, w^+)$ and $d_\Lambda(x_i, w^-)$ are the distances between the sample vector x_i and the closest correct and incorrect prototypes, respectively. We assessed the model performance by classification accuracy, true positive rate, true negative rate and macro averaged error (MAE).

While an average of symmetric matrices will be symmetric, the challenge in averaging symmetric positive definite matrices is maintaining positive definiteness. Metric tensors are expected to be symmetric and positive definite, meaning all their eigenvalues are positive. It is important to ensure the averaged matrix remains positive definite. This can be achieved by calculating the mean metric tensor on the manifold of symmetric positive definite matrices (as opposed to performing a simple average).

Further, we introduced ensemble learning, combining multiple models to make more accurate predictions, and enhance robustness. In particular, for each cross-validation, we split the data into training-fold and test-fold. To mitigate any potential biases due to class imbalance in the dataset (Clinically Stable (n = 100), Clinically Declining (n = 156)), we resampled the data to generate balanced classes. For each training-fold, we repeatedly (n = 400) randomly down-sampled the majority class (i.e. Clinically Declining) to match the size of the minority class (i.e. Clinically Stable). For each resampling, we generated a new GMLVQ model that was trained on the resampled data in the training-fold (i.e. the model learned the metric tensor and prototype locations specific to each resampled training set) and validated on the test-fold. From

this ensemble of models, we selected the top 20% (n = 80) classifiers based on their training set performance. Note that this selection could not be done based on the out-of-sample performance, as this could lead to biased models; that is, the ensemble members would be selected on the same sets the ensemble generalization performance is assessed on. However, as we employ one prototype per class, under the global metric tensor, all classification decision boundaries are linear. Hence, the risk of overfitting the training set is minimized and the training set classification accuracies are reasonable proxies for the out-of-sample ones: the ensemble member selection can be performed on the training sets without needing to compromise the out-of-sample sets. We then estimated the class balanced accuracy based on a) majority vote [12], i.e. the class label that receives the most votes from the ensemble models is selected as the final prediction, b) the average performance across the selected classifiers [13]. This ensemble learning approach with cross-validation helps mitigate for potential individual model biases, resulting in more robust and accurate predictions.

GMLVQ-Scalar Projection

Moving beyond binary classifications, we extended the GMLVQ framework to generate continuous predictions from baseline cognitive data and structural MRI data (temporal lobe GM density). In particular, we employed GMLVQ-Scalar Projection [5] that extracts distance information from the sample vector and the learnt prototypes (representing Clinically stable vs. Declining). GMLVQ-Scalar Projection measures the distance in the learnt space, after applying the learnt metric tensor, between an individual and the prototype representing Clinically Stable along the direction separating Clinically Stable vs. Declining (the line connecting stable and progressive class prototypes). We extracted the scalar projection using the average prototypes and metric tensors of the selected top 20% classifiers to capture robust information across the ensemble of trained classifiers (note, majority voting does not support extraction of scalar projection), resulting in similar performance as majority voting (Table S1).

In particular, following the learning process in GMLVQ, we transformed the sample vector x and prototypes $w_{(stable, progressive)}$ into the learned space using the metric tensor Λ. The geometric effect of the metric tensor on the original data vectors x can be interpreted as change of basis and rescaling: $X_i = \Lambda^{1/2} x_i$. Under such transformation, the learnt distances between data points x_i are equal to the usual Euclidean distance between the transformed points X_i. Hence, we accordingly transformed the data points and class prototypes: $W_{(stable,progressive)} = \Lambda^{1/2}$ $w_{(stable,progressive)}$.

To further analyze the separation of each vector x from the prototype W along the given direction, we centered the coordinate system on the prototype W_{stable} and calculated the orthogonal projection of each vector x onto the direction vector. The direction vector is defined as the difference between the stable and the progressive prototypes:

$$Projection = \frac{\overrightarrow{X_l W_{stable}} \cdot \overrightarrow{W_{progressive} W_{stable}}}{\left|\overrightarrow{W_{progressive} W_{stable}}\right|}$$

To normalize the projections with respect to the position of the prototype $W_{progressive}$, we divided each projection by the norm of the direction vector. This normalization step allows us to determine the relative separation of a test point from the stable prototype.

$$Scalar\ Projection = \frac{\overrightarrow{X_l W_{stable}} \cdot \overrightarrow{W_{progressive} W_{stable}}}{\left|\overrightarrow{W_{progressive} W_{stable}}\right|^2}$$

The resulting value indicates the separation of a test point from the prototype W_{stable} along the direction of $\overrightarrow{W_{progressive} W_{stable}}$. A large positive value suggests a significant separation from the stable prototype W_{stable}in the direction, while a large negative value indicates a substantial separation in the opposite direction. A value of 1 signifies that a sample is incident to the prototype $W_{progressive}$, while a value of 0 indicates that a sample is incident to the prototype W_{stable}, representing the stable class. The decision boundary separating the two classes within the binary classification framework is located at a value of 0.5. The scalar projection, obtained

by performing these calculations, yields a large positive value for Clinically Declining individuals and a zero or negative value for Clinically Stable individuals. This scalar projection index serves as a discriminative indicator of the classification task, where higher positive values correspond to a higher likelihood of being classified as Clinically Declining. That is, the scalar projection index captures information about how far an individual is from the Clinically Stable prototype, serving as an individualized PPM-derived prognostic index. We have previously shown that this index relates significantly to the rate of memory decline, allowing us to estimate how fast an individual progresses from Clinically Stable to AD [1,5].

Non-parametric Statistical Analyses

Treatment outcomes based on PPM-stratification: To account for deviations from normality for the PPM-derived index data (Figure S2), we repeated the statistical analysis using Analysis of Covariance (ANCOVA), to test Treatment (placebo, 20 mg, 50mg) effects on outcomes (β-Amyloid and cognitive scores: CDR-SOB and ADAS-Cog13) across timepoints (week 1, 52, 104) for each PPM-stratified group (Slow vs. Rapid progressive).

Treatment effects on β-Amyloid: Consistent with the MMRM analysis, ANCOVA analysis including fixed effects for treatment, timepoint, PPM-stratified group showed significant main effect of Timepoint ($F(2, 840) = 61.21$, $p < 0.001$) and PPM-stratified group ($F(1, 840) = 704.95$, $p < 0.001$), significant interactions for Treatment × Timepoint ($F(4, 840) = 15.49$, $p < 0.001$) and PPM-stratified group x Timepoint ($F(2, 840) = 9.06$, $p < 0.001$). Post-hoc comparisons showed that this PPM-stratified group x Timepoint interaction was significant for the lanabecestat 20mg ($F(2, 302) = 5.72$, $p < 0.01$) and lanabecestat 50mg ($F(2, 250) = 3.77$, $p = 0.02$) but not the placebo ($F(2, 261) = 1.33$, $p = 0.27$) group.

Treatment effects on CDR-SOB: Consistent with the MMRM analysis, ANCOVA analysis including fixed effects for treatment, timepoint, PPM-stratified group showed: a) significant

interactions: PPM-stratified group x Treatment x Timepoint ($F(4, 2850) = 2.76$, $p = 0.03$), PPM-stratified group x Timepoint ($F(2, 2850) = 17.29$, $p < 0.001$); b) significant main effects of PPM-stratified group ($F(1, 2850) = 176.36$, $p < 0.001$), Treatment ($F(2, 2850) = 5.43$, $p < 0.01$), Timepoint ($F(2, 2850) = 462.77$, $p < 0.001$). Post-hoc comparisons showed that PPM-stratified group x Timepoint interaction was significant for the lanabecestat 20mg ($F(2, 939) = 4.86$, $p < 0.01$) and lanabecestat 50mg ($F(2, 921) = 18.84$, $p <0.001$) but not the placebo ($F(2, 958) = 1.44$, $p = 0.23$) group.

Treatment effects on Alzheimer's Disease Assessment Scale - Cognitive subscale 13 (ADAS-Cog13): Consistent with the MMRM analysis, ANCOVA analysis including fixed effects for treatment, timepoint, PPM-stratified group showed a significant PPM-stratified group x Timepoint interaction ($F(2, 2823) = 10.58$, $p < 0.0001$).

Supplementary Figures

Figure S1

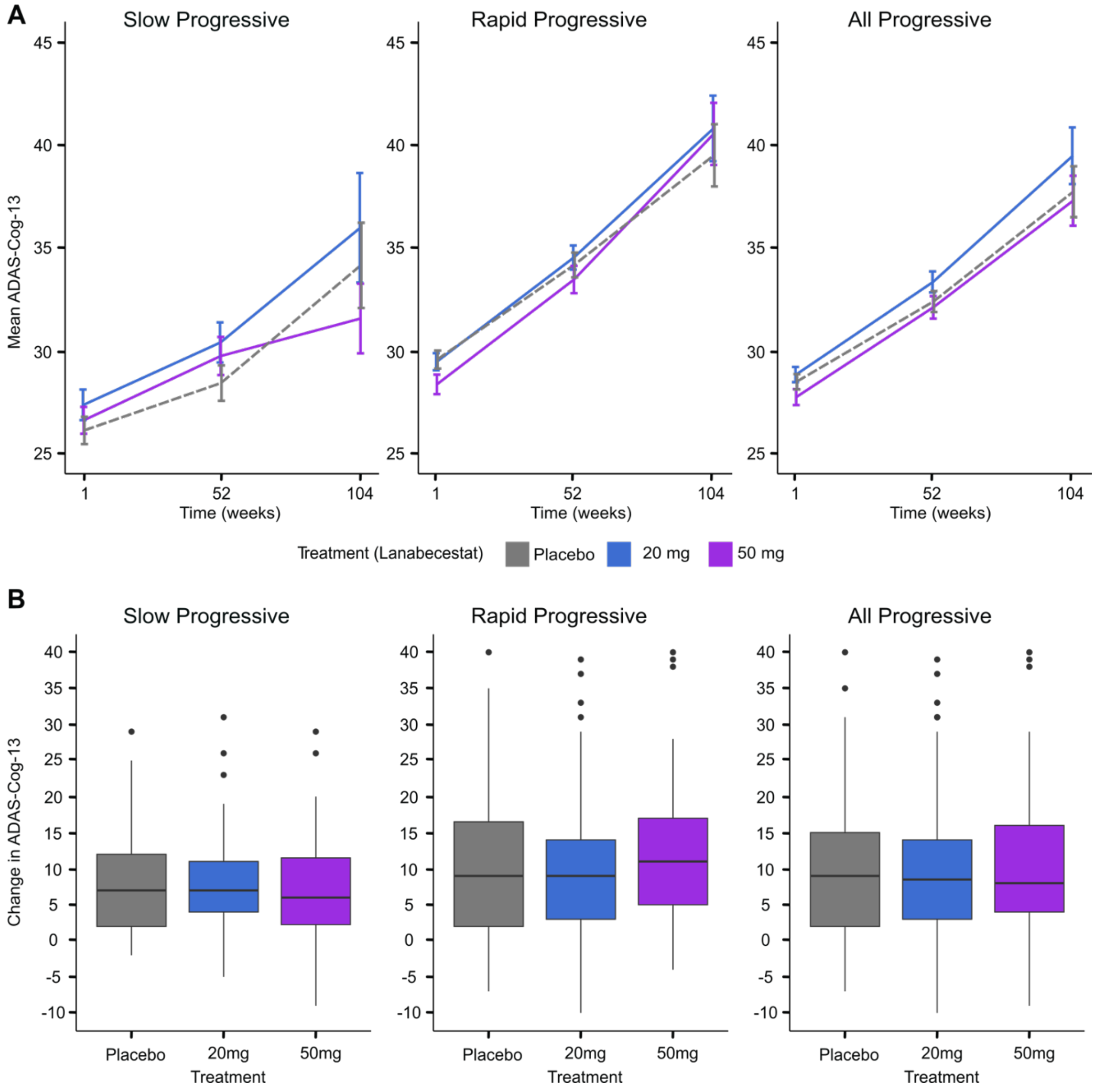

Figure S1: Treatment effects of lanabecestat in the AMARANTH trial on ADAS-Cog 13 **A.** Mean ADAS-Cog13 scores over time for Slow, Rapid, and All Progressive individuals (sample size: Table S3) in the Placebo (grey dashed), lanabecestat 20mg (blue), and lanabecestat 50mg (purple) treatment groups. Error bars indicate the standard error of the mean across individuals (SEM). B. Box plots of change in ADAS-Cog13 (week 104 minus week 1) for Slow, Rapid, and All Progressive (sample size: Table S3) showing— similar with CDR-SOB, albeit not significant— reduction in cognitive decline for the slow progressive individuals in the 50mg treatment group vs. placebo. Black lines in the box plots indicate the median for placebo (grey), lanabecestat 20mg (blue), and lanabecestat 50mg (purple), solid black box represents the 25th to 75th percentile, the black vertical lines represent the range of the data, black circles indicate outliers. Source data are provided as a Source Data file.

Figure S2

A

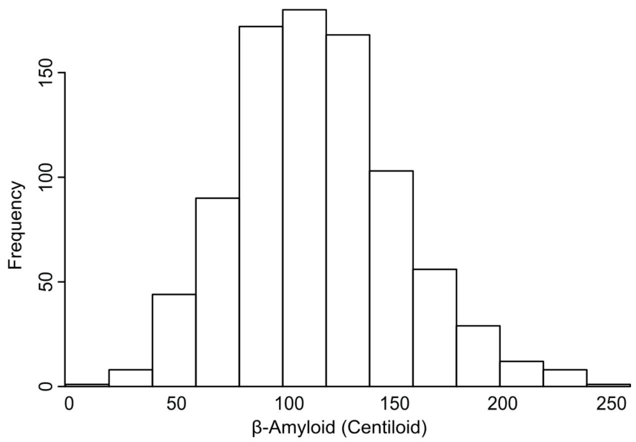

B

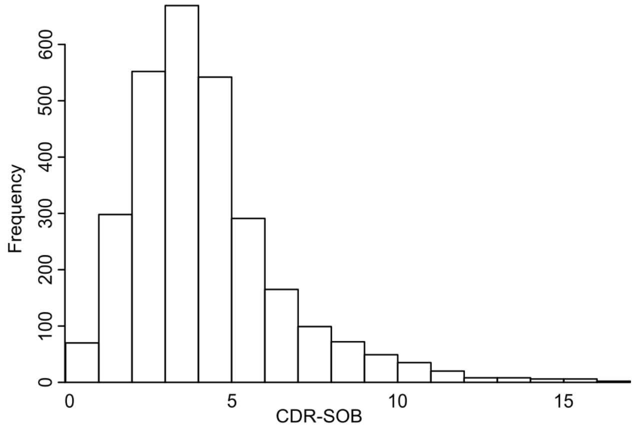

C

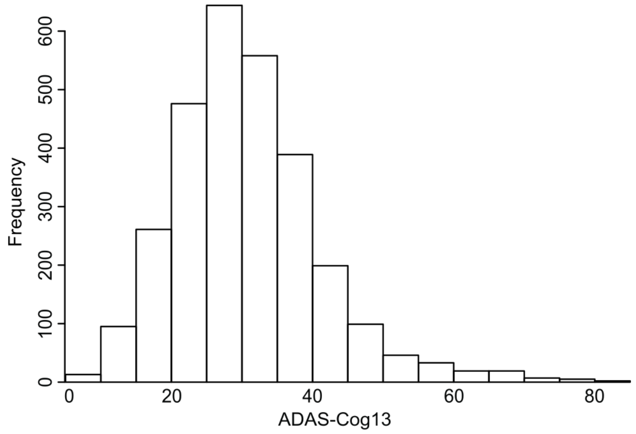

Figure S2. Data Distributions for: A. β-Amyloid in centiloids (Shapiro-Wilk test, $W = 0.99$, $p < 0.001$), B. CDR-SOB (Shapiro-Wilk test, $W = 0.89$, $p < 0.001$), C. ADAS-Cog13 (Shapiro-Wilk test, $W = 0.95$, $p < 0.001$).

Supplementary Tables

Table S1. PPM class-balanced accuracy, sensitivity, specificity, AUC, F1-score, precision, Recall, for Clinically Stable vs. Clinically declining classification for ADNI training data (10 repetitions x 10-fold cross-validation).

**Performance Majority voting**	**Performance Average of classifiers**
Accuracy = 91.1%<br>AUC: 0.94<br>sensitivity = 87.5%<br>specificity = 94.2%<br>Precision = 93.8%<br>F1 score: 90.5%	accuracy = 90.6%<br>AUC = 0.94<br>sensitivity = 87.1%<br>specificity = 94.1%<br>Precision = 95.7%<br>F1 score = 91.2 %

Table S2. AMARANTH sample size for β-Amyloid per treatment, PPM-derived group and timepoint (week 1, 52, 104).

	**Rapid progressive**			**Slow progressive**		
Week	*1*	*52*	*104*	*1*	*52*	*104*
**Placebo**	96	20	76	44	8	36
**20mg**	114	34	80	45	14	31
**50mg**	81	22	59	54	16	38
**Sum**	291	76	215	143	38	105

Table S3. AMARANTH sample size for: **A.** CDR-SOB, **B.** ADAS-Cog13 per treatment, PPM-derived group and timepoint (week 1, 52, 104).

**A.**	**Rapid progressive**			**Slow progressive**		
Week	*1*	*52*	*104*	*1*	*52*	*104*
**Placebo**	312	269	90	142	120	47
**20mg**	313	270	99	133	110	36
**50mg**	294	239	71	160	138	41
**Sum**	919	778	260	435	368	124

**B.**	**Rapid progressive**			**Slow progressive**		
Week	*1*	*52*	*104*	*1*	*52*	*104*
**Placebo**	311	267	86	142	119	43
**20mg**	313	266	87	133	108	32
**50mg**	292	232	66	135	138	38
**Sum**	916	765	239	410	365	113

Table S4 : Statistical analyses (MMRM) for all outcomes

Measure / Factor	**Timepoint**	**Treatment**	**PPM-stratified group**	**Treatment × Timepoint**	**PPM-stratified group x Treatment**	**PPM-stratified group x Timepoint**	**PPM-stratified group x Treatment x Timepoint**
**β-Amyloid**	F(2, 485.43) = 37.01, **p < 0.001**	F(2, 493.35) = 2.65, p = 0.07	F(1, 489.19) = 84.04, **p < 0.001**	F(4, 484.87) = 10.27, **p < 0.001**	F(2, 492.95) = 0.83, p = 0.43	F(2, 485.46) = 8.38, **p < 0.001**	F(4, 485.01) = 0.86, p < 0.49
**CDR-SOB**	F(2, 2190.7) = 24.08, **p < 0.001**	F(2, 1870.5) = 3.00., **p = 0.05**	F(1, 1809.1) = 35.70, **p < 0.001**	F(4, 2172.8) = 2.20, p = 0.067	F(2, 1866.4) = 2.51, p = 0.08	F(2, 2163.7) = 14.72, **p < 0.001**	F(4, 2173.3) = 2.62, **p < 0.05**
ADAS-Cog13	F(2, 2152.3) = 24.42, **p < 0.001**	F(2, 1903.7) = 0.93, p < 0.39	F(1, 1848.9) = 29.51, **p < 0.001**	F(4, 2157.8) = 0.94, p = 0.44	F(2, 1903.3) = 0.35, p = 0.71	F(2, 2162.8) = 10.37, **p < 0.001**	F(4, 2157.9) = 1.17, p = 0.32

Factor	**Post hoc comparisons: PPM-stratified group x Timepoint**		
	Placebo	**Lanabecestat 20mg**	**Lanabecestat 50mg**
**β-Amyloid**	F(2, 152.76) = 1.20 p= 0.30	F(2, 184.22) = 5.36, p< **0.01**	F(2, 146.80) = 3.47, p= **0.03**
**CDR-SOB**	F(2, 720.26) = 0.96, p= 0.38	F(2, 716.64) = 4.301, **p = 0.014**	F(2, 709.67) = 15.28, **p < 0.001**
ADAS-Cog13	F(2, 720.14) = 4.53, **p=0.011**	F(2, 719.95) = 3.31, **p = 0.03**	F(2, 706.74) = 6.83, **p < 0.001**

Table S5. Least Squares Mean from MMRM models per outcome.

**Outcome measure**	**Group**	**Placebo**	**Lanabecestat 20mg**	**Lanabecestat 50mg**
β-Amyloid	Trial sample	-2.08 (1.86)	-15.76 (1.89)	-19.74 (1.97)
	All progressive	-0.114 (2.38)	-15.32 (2.46)	-19.67 (2.43)
	Slow progressive	3.48 (3.21)	-8.53 (3.43)	-14.04 (3.10)
	Rapid progressive	-3.71 (2.90)	-22.15 (2.78)	-25.38 (3.28)
CDR-SOB	Trial sample	3.02 (0.17)	3.17 (0.17)	3.17 (0.18)
	All progressive	2.51 (0.15)	2.52 (0.16)	1.97 (0.16)
	Slow progressive	2.21 (0.24)	1.90 (0.27)	1.03 (0.25)
	Rapid progressive	2.74 (0.18)	3.04 (0.17)	2.86 (0.20)
ADAS-Cog13	Trial sample	10.31 (0.55)	9.38 (0.56)	10.72 (0.58)
	All progressive	10.0 (0.55)	9.4 (0.59)	10.2 (0.59)
	Slow progressive	9.31 (0.85)	8.36 (0.95)	7.92 (0.89)
	Rapid progressive	10.4 (0.65)	10.1 (0.65)	12.0 (0.73)

References

1. Giorgio, J. *et al.* A robust and interpretable machine learning approach using multimodal biological data to predict future pathological tau accumulation. *Nat Commun* **13**, (2022).
2. Wessels, A. M. *et al.* Efficacy and Safety of Lanabecestat for Treatment of Early and Mild Alzheimer Disease: The AMARANTH and DAYBREAK-ALZ Randomized Clinical Trials. *JAMA Neurol* **77**, 199–209 (2020).
3. Kueper, J. K., Speechley, M. & Montero-Odasso, M. The Alzheimer's Disease Assessment Scale–Cognitive Subscale (ADAS-Cog): Modifications and Responsiveness in Pre-Dementia Populations. A Narrative Review. *Journal of Alzheimer's Disease* **63**, 423 (2018).
4. Wessels, A. M. *et al.* Efficacy and Safety of Lanabecestat for Treatment of Early and Mild Alzheimer Disease: The AMARANTH and DAYBREAK-ALZ Randomized Clinical Trials. *JAMA Neurol* **77**, 199–209 (2020).
5. Giorgio, J., Landau, S. M., Jagust, W. J., Tino, P. & Kourtzi, Z. Modelling prognostic trajectories of cognitive decline due to Alzheimer's disease. *Neuroimage Clin* **26**, (2020).
6. Ashburner, J. A fast diffeomorphic image registration algorithm. *Neuroimage* **38**, 95–113 (2007).
7. Landau, S. M. *et al.* Measurement of longitudinal β-amyloid change with 18F-florbetapir PET and standardized uptake value ratios. *J Nucl Med* **56**, 567–574 (2015).
8. Klunk, W. E. *et al.* The Centiloid Project: Standardizing quantitative amyloid plaque estimation by PET. *Alzheimer's & Dementia* **11**, 1-15.e4 (2015).
9. Joshi, A. D. *et al.* Performance Characteristics of Amyloid PET with Florbetapir F 18 in Patients with Alzheimer's Disease and Cognitively Normal Subjects. *Journal of Nuclear Medicine* **53**, 378–384 (2012).
10. Harrison, T. M. *et al.* Longitudinal tau accumulation and atrophy in aging and alzheimer disease. *Ann Neurol* **85**, 229–240 (2019).
11. Clark, C. M. *et al.* Cerebral PET with florbetapir compared with neuropathology at autopsy for detection of neuritic amyloid-β plaques: a prospective cohort study. *Lancet Neurol* **11**, 669–678 (2012).
12. Lam, L. & Suen, S. Y. Application of majority voting to pattern recognition: an analysis of its behavior and performance. *IEEE Transactions on Systems, Man, and Cybernetics - Part A: Systems and Humans* **27**, 553–568 (1997).
13. Kuncheva, L. I. *Combining Pattern Classifiers: Methods and Algorithms*. (John Wiley & Sons, 2014).
