# Supplementary material for: AI-guided patient stratification improves outcomes and efficiency in the AMARANTH Alzheimer’s Disease clinical trial
Source: Nat Commun. 2025 Jul 17;16:6244. doi: 10.1038/s41467-025-61355-3 (PMC12271323; doi:10.1038/s41467-025-61355-3)
Supplement: Supplementary file 2 — Reporting Summary [file 41467_2025_61355_MOESM2_ESM.pdf]

nature portfolio

Corresponding author(s): Zoe Kourtzi

Last updated by author(s): 25/03/25

Reporting Summary

Nature Portfolio wishes to improve the reproducibility of the work that we publish. This form provides structure for consistency and transparency in reporting. For further information on Nature Portfolio policies, see our Editorial Policies and the Editorial Policy Checklist.

Statistics

For all statistical analyses, confirm that the following items are present in the figure legend, table legend, main text, or Methods section.

n/a	Confirmed	
☐	☒	The exact sample size (*n*) for each experimental group/condition, given as a discrete number and unit of measurement
☐	☒	A statement on whether measurements were taken from distinct samples or whether the same sample was measured repeatedly
☐	☒	The statistical test(s) used AND whether they are one- or two-sided<br>*Only common tests should be described solely by name; describe more complex techniques in the Methods section.*
☐	☒	A description of all covariates tested
☐	☒	A description of any assumptions or corrections, such as tests of normality and adjustment for multiple comparisons
☐	☒	A full description of the statistical parameters including central tendency (e.g. means) or other basic estimates (e.g. regression coefficient) AND variation (e.g. standard deviation) or associated estimates of uncertainty (e.g. confidence intervals)
☐	☒	For null hypothesis testing, the test statistic (e.g. *F*, *t*, *r*) with confidence intervals, effect sizes, degrees of freedom and *P* value noted<br>*Give P values as exact values whenever suitable.*
☒	☐	For Bayesian analysis, information on the choice of priors and Markov chain Monte Carlo settings
☒	☐	For hierarchical and complex designs, identification of the appropriate level for tests and full reporting of outcomes
☐	☒	Estimates of effect sizes (e.g. Cohen's *d*, Pearson's *r*), indicating how they were calculated

Our web collection on statistics for biologists contains articles on many of the points above.

Software and code

Policy information about availability of computer code

Data collection	no software was used
Data analysis	Matlab, Python, SPSS; Code is available in the Univ of Cambridge repository: https://doi.org/10.17863/CAM.117732

For manuscripts utilizing custom algorithms or software that are central to the research but not yet described in published literature, software must be made available to editors and reviewers. We strongly encourage code deposition in a community repository (e.g. GitHub). See the Nature Portfolio guidelines for submitting code & software for further information.

Data

Policy information about availability of data

All manuscripts must include a data availability statement. This statement should provide the following information, where applicable:

- Accession codes, unique identifiers, or web links for publicly available datasets
- A description of any restrictions on data availability
- For clinical datasets or third party data, please ensure that the statement adheres to our policy

Data may be obtained in accordance with AstraZeneca's data sharing policy described at: https://astrazenecagrouptrials.pharmacm.com/ST/Submission/Disclosure. The raw trial data are protected and are not available due to data privacy laws. Anonymized processed data for studies directly listed on Vivli can be requested through Vivli at www.vivli.org. AstraZeneca Vivli member page is available outlining further details: https://vivli.org/ourmember/astrazeneca/. Data not listed on Vivli could be requested through Vivli at https://vivli.org/members/enquiries-aboutstudies-not-listed-on-the-vivli-platform/. Source data for figures are provided with this paper and are available at the Cambridge University repository: https://doi.org/10.17863/CAM.117732

Research involving human participants, their data, or biological material

Policy information about studies with human participants or human data. See also policy information about sex, gender (identity/presentation), and sexual orientation and race, ethnicity and racism.

Reporting on sex and gender	In AMARANTH (n = 1354, placebo: 53.8% female, 20mg: 53.5% female, 50mg: 52% female) eligible patients were randomized 1:1:1 to once-daily oral doses of 20mg lanabecestat, 50mg lanabecestat, or placebo, stratified by disease status at baseline (MCI attributable to AD or mild AD). Across treatment groups, patient baseline demographics were well balanced with regard to sex, age, race, educational level.<br>For the modeling, we regressed out age, sex, and education from the training model features to account for potential confounding differences in these covariates across classes.
Reporting on race, ethnicity, or other socially relevant groupings	see: see: Wessels, A. M. et al. Efficacy and Safety of Lanabecestat for Treatment of Early and Mild Alzheimer Disease: The AMARANTH and DAYBREAK-ALZ Randomized Clinical Trials. JAMA Neurol 77, 199–209 (2020).<br>Baseline demographics and clinical baseline characteris- tics are summarized in Table 2.
Population characteristics	Male or Female, aged 55 to 85 years inclusive at the time of signing the consent form.<br>Patients with mild AD dementia were eligible to be included in the study only if they met all of the following criteria at screening.
Recruitment	Our study reanalysed data samples from a published clinical trial, it did not recruit participants. For more details on the AMARANTH clinical trial please see: Wessels, A. M. et al. Efficacy and Safety of Lanabecestat for Treatment of Early and Mild Alzheimer Disease: The AMARANTH and DAYBREAK-ALZ Randomized Clinical Trials. JAMA Neurol 77, 199–209 (2020).
Ethics oversight	The protocol, patient information, consent form, and other relevant study documentation were approved by the ethics committees or institutional review boards of each trial site before study initiation. The study was conducted in accordance with ethical principles originating from the Declaration of Helsinki and was consistent with good clinical practice and applicable regulatory requirements.

Note that full information on the approval of the study protocol must also be provided in the manuscript.

Field-specific reporting

Please select the one below that is the best fit for your research. If you are not sure, read the appropriate sections before making your selection.

☒ Life sciences ☐ Behavioural & social sciences ☐ Ecological, evolutionary & environmental sciences

For a reference copy of the document with all sections, see nature.com/documents/nr-reporting-summary-flat.pdf

Life sciences study design

All studies must disclose on these points even when the disclosure is negative.

Sample size	see: Wessels, A. M. et al. Efficacy and Safety of Lanabecestat for Treatment of Early and Mild Alzheimer Disease: The AMARANTH and DAYBREAK-ALZ Randomized Clinical Trials. JAMA Neurol 77, 199–209 (2020).
Data exclusions	see Table S2 , Table S3
Replication	10-fold cross validation
Randomization	see: Wessels, A. M. et al. Efficacy and Safety of Lanabecestat for Treatment of Early and Mild Alzheimer Disease: The AMARANTH and DAYBREAK-ALZ Randomized Clinical Trials. JAMA Neurol 77, 199–209 (2020).

Blinding	see: Wessels, A. M. et al. Efficacy and Safety of Lanabecestat for Treatment of Early and Mild Alzheimer Disease: The AMARANTH and DAYBREAK-ALZ Randomized Clinical Trials. JAMA Neurol 77, 199–209 (2020).

Reporting for specific materials, systems and methods

We require information from authors about some types of materials, experimental systems and methods used in many studies. Here, indicate whether each material, system or method listed is relevant to your study. If you are not sure if a list item applies to your research, read the appropriate section before selecting a response.

Materials & experimental systems

n/a	Involved in the study
☒	☐ Antibodies
☒	☐ Eukaryotic cell lines
☒	☐ Palaeontology and archaeology
☒	☐ Animals and other organisms
☐	☒ Clinical data
☒	☐ Dual use research of concern
☒	☐ Plants

Methods

n/a	Involved in the study
☒	☐ ChIP-seq
☒	☐ Flow cytometry
☐	☒ MRI-based neuroimaging

Clinical data

Policy information about clinical studies

All manuscripts should comply with the ICMJEguidelines for publication of clinical research and a completedCONSORT checklist must be included with all submissions.

Clinical trial registration	NCT02245737
Study protocol	see: Wessels, A. M. et al. Efficacy and Safety of Lanabecestat for Treatment of Early and Mild Alzheimer Disease: The AMARANTH and DAYBREAK-ALZ Randomized Clinical Trials. JAMA Neurol 77, 199–209 (2020).
Data collection	see: Wessels, A. M. et al. Efficacy and Safety of Lanabecestat for Treatment of Early and Mild Alzheimer Disease: The AMARANTH and DAYBREAK-ALZ Randomized Clinical Trials. JAMA Neurol 77, 199–209 (2020).
Outcomes	see: Wessels, A. M. et al. Efficacy and Safety of Lanabecestat for Treatment of Early and Mild Alzheimer Disease: The AMARANTH and DAYBREAK-ALZ Randomized Clinical Trials. JAMA Neurol 77, 199–209 (2020).

Plants

Seed stocks	n/a
Novel plant genotypes	n/a
Authentication	n/a

Magnetic resonance imaging

Experimental design

Design type	structural MRI
Design specifications	n/a
Behavioral performance measures	n/a

Acquisition

Imaging type(s)	structural
Field strength	Structural MRIs for the AMARANATH samples were acquired at different sites among Australia, Belgium, Canada, USA,

France, Germany, United Kingdom, Italy, Japan, Poland equipped with 1.5T and 3T MRI scanners ('Philips', 'Siemens', 'GE', 'DicomCleaner', 'Ingenia') using a 3D T1-weighted sequences.

Sequence & imaging parameters: 3D T1-weighted sequences.

Area of acquisition: whole brain

Diffusion MRI: ☐ Used ☒ Not used

Preprocessing

Preprocessing software: All imaging pre-processing was performed using Statistical Parametric Mapping 12 in MATLAB (SPM12, http://www.fil.ion.ucl.ac.uk/spm/) following our previously published pipeline

Normalization: Structural images were reoriented and segmented into grey matter, white matter and cerebrospinal fluid. We used the DARTEL toolbox 6 to generate a study-specific template to which all scans were normalised. Individual grey matter segmentation volumes were normalised to MNI space without modulation. The unmodulated values for each voxel represent grey matter density at the voxel location.

Normalization template: MNI

Noise and artifact removal: All images were smoothed using a 3mm3 isotropic kernel and resliced to MNI resolution 1.5 × 1.5 × 1.5 mm voxel size.

Volume censoring: n/a

Statistical modeling & inference

Model type and settings: We generated an index of medial temporal grey matter density (MTL GM density). We first performed feature construction in the whole brain T1-weighted MRI voxel space using partial least squares regression with recursive feature elimination (PLSr-RFE) on ADNI (ADNI-GO ADNI-2) data. In particular, we tested for grey matter voxels that predicted memory decline (i.e. annualized change in ADNI memory composite), iteratively removing predictors (voxels) that had weak predictive values and resulting in a bilateral cluster of voxels in MTL that predict cognitive decline. That is, this reduced set of new orthogonal features span the voxel subspace that maximises covariance with the relevant response variable (i.e. memory decline). Using this method, we determined an ROI defined by a matrix of voxel weights in the medial temporal lobe and extracted grey matter density.

Effect(s) tested: n/a

Specify type of analysis: ☒ Whole brain ☐ ROI-based ☐ Both

Statistic type for inference (See Eklund et al. 2016): voxel-wise

Correction: FDR

Models & analysis

n/a	Involved in the study
☒	☐ Functional and/or effective connectivity
☒	☐ Graph analysis
☒	☐ Multivariate modeling or predictive analysis
